# Tremor Syndromes: An Updated Review

**DOI:** 10.3389/fneur.2021.684835

**Published:** 2021-07-26

**Authors:** Abhishek Lenka, Joseph Jankovic

**Affiliations:** ^1^Department of Neurology, Medstar Georgetown University Hospital, Washington, DC, United States; ^2^Parkinson's Disease Center and Movement Disorders Clinic, Department of Neurology, Baylor College of Medicine, Houston, TX, United States

**Keywords:** tremor, essential tremor plus, action tremor, rest tremor, dystonic tremor, neuropathic tremor, myorhythmia, orthostatic tremor

## Abstract

Tremor is the most commonly encountered movement disorder in clinical practice. A wide range of pathologies may manifest with tremor either as a presenting or predominant symptom. Considering the marked etiological and phenomenological heterogeneity, it would be desirable to develop a classification of tremors that reflects their underlying pathophysiology. The tremor task force of the International Parkinson Disease and Movement Disorders Society has worked toward this goal and proposed a new classification system. This system has remained a prime topic of scientific communications on tremor in recent times. The new classification is based on two axes: 1. based on the clinical features, history, and tremor characteristics and 2. based on the etiology of tremor. In this article, we discuss the key aspects of the new classification, review various tremor syndromes, highlight some of the controversies in the field of tremor, and share the potential future perspectives.

## Introduction

Tremor is an involuntary, rhythmic, and oscillatory movement which may involve one or several body parts (1, 2). After leg stereotypy syndrome (3), tremor is the most commonly observed movement disorder in adults (4, 5). Tremor can be an isolated manifestation of a disease such as essential tremor (ET) or it can be a part of other neurological disorders. The task force on tremor of the International Parkinson and Movement Disorders Society (IPMDS) proposed a classification scheme based on two axes; axis 1- emphasizing the clinical features, history, and tremor characteristics and axis 2- emphasizing the potential etiologies of tremor (1). One of the major aims was to redefine ET (“bilateral upper limb action tremor” of “at least 3 years' duration”) and to introduce the concept of ET plus (ET with additional neurologic soft signs such as dystonia, ataxia, parkinsonism) (1). The publication engendered a great deal of controversy about the definition of ET and related syndromes. Since tremor has a vastly heterogeneous etiological spectrum, it is important to fully appreciate the phenomenology of tremor in various tremor syndromes and other neurological features associated with those syndromes.

The major objective of this article is to provide an updated review of various tremor syndromes with special reference to the new bi-axial classification system. We also highlight some of the controversies in the field of tremor, and share our perspectives for the future research.

## Methods

For this narrative review, the literature search in PubMed was done in January-April 2021. A broad search strategy was used with several keywords and combinations related to tremor (“Tremor,” “Tremor syndrome,” “Essential tremor,” “Action tremor,” “Rest tremor,” “Intention tremor,” “Postural tremor,” “Kinetic tremor,” “Isometric tremor,” “Task-specific tremor,” “Focal tremor,” “Palatal tremor,” “Tremor AND genetics,” “Tremor AND etiology,” “Tremor AND neurodegeneration,” “Tremor AND Toxins,” “Tremor AND Neuropathy.” Titles and abstracts were reviewed and when appropriate from the standpoint of the theme of the current review topic, articles were shortlisted, reviewed in detail, and used for the references.

## Types of Tremor Based on the Activation Pattern

**Figure 1:**
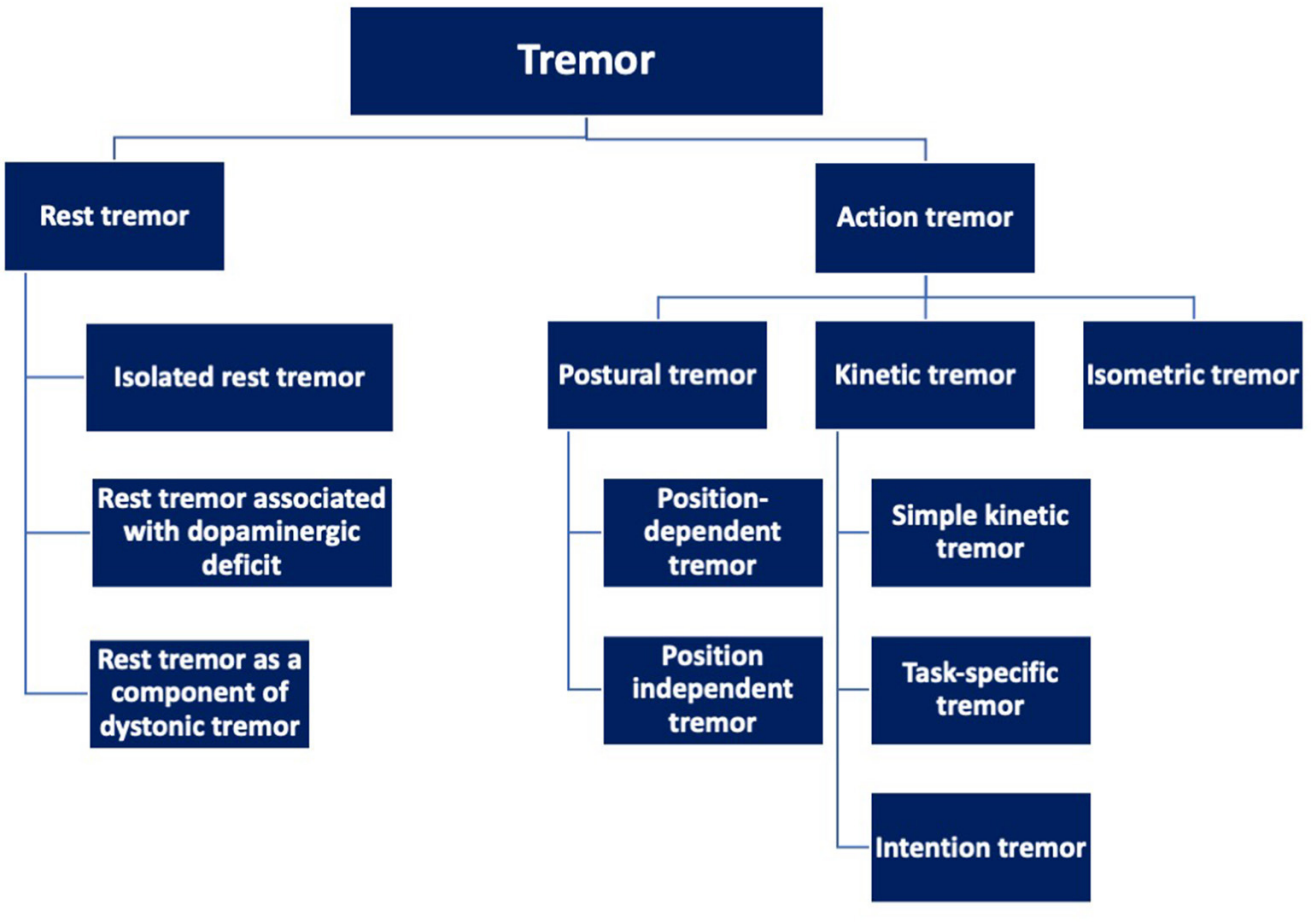
Categories of tremor based on the activation pattern.

Based on the activation pattern, tremor is broadly categorized into rest tremor or action tremor (Figure 1) (1). As evident from the name, action tremor manifests only during any activity. It is further divided into postural tremor, kinetic tremor, and isometric tremor. Postural tremor may occur in specific positions (position-dependent tremor) or may occur independently of any specific position (position-independent tremor). Kinetic tremor is further divided into simple kinetic tremor (non-specific to any activity), task-specific tremor (while doing a specific task- writing, playing musical instruments, etc), and intention tremor (while performing goal-directed activities such as finger-to-nose test). Isometric tremor occurs during sustained muscle contraction without any gross movement of the body part other than the tremor (Table 1).

**Table 1:** Tremor syndromes based on the predominant manifestation of the tremor (Axis-1).

**Tremor category based on activation/position**	**Tremor subcategories**	**Key features**
Action/rest tremor	Essential tremor	Body parts involved: Bilateral upper extremities involvement for 3-years is mandatory for diagnosis. Voice, head, lower extremities may be involved. Key features: 4–12 Hz action tremor
	Essential tremor plus	Tremor fulfilling the criteria of ET along with additional neurological signs (dystonia, rest tremor, impaired tandem gait)
	Enhanced physiologic tremor	Body parts involved: Bilateral hands and fingers Key features: Low amplitude, high frequency tremor (8–12H z). Can be precipitated or exacerbated by anxiety, caffeine, and hypermetabolic states.
	Isolated action or rest tremor syndromes	Additional clinical features in axis-1 should be explored to reach at the diagnosis. Isolated rest tremor usually affects the upper extremities and may evolve into Parkinson's disease.
Focal tremor	Voice tremor	Body parts involved: Vocal cord, larynx, oropharynx, palate, tongue, lip) Key features: Frequency range 3.8–5.5 Hz
	Head tremor	Body parts involved: Head/neck Key features: Yes-yes or no-no or diagonal direction of tremor, often associated with cervical dystonia or essential tremor
	Palatal tremor	Body part involved: Soft palate (may be associated with myorhythmia in other body parts) Key features: Rhythmic, 0.5–5Hz tremor, may be present during sleep, may be associated with audible clicks
Task specific tremor	Primary writing tremor	Body part involved: Hand used for writing Key features: Tremor only while writing (type-A) or while adopting the hand in writing position (type-B)
	Other tremors in musicians and sports persons	Body part involved: Hand used for the specific task Key features: may be associated with focal dystonia and a compensatory posture
Orthostatic tremor	Primary orthostatic tremor	Body part involved: legs and trunk Key features: 13–18Hz, low amplitude tremor only while standing, associated with subjective unsteadiness
	Pseudo orthostatic tremor	Body part involved: legs Key features: <13 Hz low amplitude tremor, only while standing, associated with subjective unsteadiness
Tremor with additional prominent neurological signs	Re-emergent tremor	Body parts involved: Upper extremities, rarely tongue Key features: Form of postural tremor (3–5 Hz) which emerges after a latency of a few seconds when hands are kept in an anti-gravity posture. Typically present in Parkinson's disease
	Dystonic tremor	Body parts involved: Any of the body parts with dystonia Key features: Irregular, jerky tremor; worsens while resisting dystonic pull and subsides or resolves in maximal dystonic posture (“null point”).
	Holmes' tremor	Body parts involved: Bilateral upper extremity Key features: Present at rest, worsens when holding a posture, intensifies during action
	Myorhythmia	Body parts involved: Cranio-facial and limb muscles Key features: Slow, rhythmic, repetitive movements (1–4 Hz); associated with lesions of the brainstem and/or diencephalic structures
	Wing-beating tremor	Body parts involved: Upper extremities Key features: High amplitude proximal tremor when arms are in abducted position; may be present in Wilson's disease or cerebellar-outflow pathways
Others	Functional tremor	Body parts involved: Any the body part Key features: Abrupt onset, variable in frequency and amplitude, distractible, entrainable; incongruous with organic tremors.

These tremors have marked etiological heterogeneity and the tremor task force of IPMDS recommends searching for those etiologies as noted in the axis-2 classification (Table 2). The following discussion largely focuses on the key aspects of various axis-1 tremor syndromes and some of the common axis-2 nosologies that may present with tremor in the background of other neurological features.

**Table 2:** A summary of common diseases/etiologies manifesting predominantly with tremor.

**Neurodegenerative**
• Parkinson's disease • Essential tremor • Corticobasal syndrome • Progressive supranuclear palsy • Multiple system atrophy
**Genetic diseases/mitochondrial diseases**
• ANO3 (Anoctamin) mutation or DYT24 • Spinocerebellar ataxia type-12, type 40 • Klinefelter syndrome • Fragile-X tremor ataxia syndrome • Hereditary chin tremor • Charcot-Marie-Tooth disease • Leigh's disease • Mitochondrial polymerase gamma mutation
**Metabolic diseases**
• Wilson's disease • Hyperthyroidism
**Drugs and toxins**
• Anti-seizure medications: Phenytoin, valproate • Beta-2 agonists • Thyroid hormone replacement • Dopamine receptor blockers: Neuroleptics, metoclopramide • Lithium • Amiodarone • Chemotherapeutic agents: Tacrolimus, vincristine, cisplatin, methotrexate • Toxins: Mercury, lead, manganese, arsenic, cyanide, carbon monoxide, naphthalene, toluene, lindane
**Neuropathic**
• Charcot-Marie-Tooth disease • Acute inflammatory polyradiculoneuropathy • Chronic inflammatory polyradiculoneuropathy • Multifocal neuropathy with conduction block • Monoclonal gammopathies
**Other causes**
Any space occupying lesions, stroke in the basal ganglia or in the cerebello-thalamo-cortical network may result in tremor, albeit along with other focal neurological deficits

## Overview of the Axis-I Tremor Syndromes

### Action and Rest Tremor

#### Essential Tremor and Essential Tremor Plus

One of the key proposals of the tremor task force was the introduction of a new definition of ET. Accordingly, ET is defined as an isolated tremor syndrome manifesting as an action tremor of bilateral upper extremities for a minimum of 3 years duration, in the absence of any other neurological signs such as parkinsonism, ataxia, or dystonia (1). This may or may not be associated with tremor involving the voice, head, and lower extremities. Previously, several neurological soft-signs such as tandem gait impairment, subtle dystonic posturing, and memory problems were considered to be in the clinical spectrum of ET. However, as per the new classification scheme, ET patients with any such neurological soft signs are now categorized as “ET plus.” The validity of this nomenclature has remained a matter of debate in recent times and we have elaborated on this issue in the latter part of this article (6, 7).

There are no prevalence studies on this newly defined “ET” or “ET plus.” However, according to the previous diagnostic criteria, ET was one of the commonly observed movement disorders among adults. Several movement disorder centers have reclassified their ET patients using the new diagnostic criteria and have reported that ET plus outnumbers the isolated (pure) ET patients after such re-classification (8–10). As mentioned above, one of the core features of ET is action tremor (kinetic > postural) of both upper extremities. Patients subsequently may develop vocal tremor, tongue tremor, head tremor, and lower extremity tremor. The usual frequency of the action tremor of the upper extremities in ET is 4–12 Hz. Postural tremor is conventionally examined by outstretching the hands in front of the body or with arms abducted at shoulders and flexed at elbows with hands held pronated in front of the chest (“wing-beating” position), whereas kinetic tremor is best evaluated during finger-nose-finger maneuver, by drawing spirals on a paper, or by pouring water between two cups (11). While the upper extremities in patients with ET have similar tremor frequencies (12), several studies, based on both objective (12) and subjective assessments (13), have reported that there may be asymmetry in tremor amplitude between the upper extremities. Although may not be universally present, alcohol responsiveness is one of the well-known characteristics of tremor in ET patients (14). While alcohol responsiveness and family history have been traditionally considered important features of ET, these were not included in the definition of ET according to the “consensus statement” (1). Tremor in ET may be difficult to differentiate from dystonic hand tremor, especially when the dystonia is subtle. In such cases, certain clinical clues that include irregularity of tremor with jerky component, abnormal hand posturing, sensory trick, null point phenomenon, and lack of a clear axis while drawing spirals may be helpful as these are commonly observed in dystonic tremor (15).

Tremor in ET patients tends to worsen over time in terms of severity as well as in the number of body parts involved and, as discussed below, may become associated with parkinsonism, dystonia, ataxia and other motor disorders (16). In addition to tremor, patients with ET may develop several non-motor symptoms (NMS) such as cognitive impairment, anxiety, depression, apathy, and sleep disturbances (17). Hence, neurologists should evaluate all ET patients for both motor and NMS.

There is growing body of evidence that some ET patients when followed longitudinally develop PD (10, 18, 19). Based on many clinical, epidemiologic, imaging, genetic and pathologic studies, a subset of ET patients appears to be at a high risk of developing PD. Besides PD with antecedent ET, ET may follow the onset of PD (ET with antecedent PD). These ET-PD patients seem to have a slower progression and more favorable prognosis than PD in general, similar to tremor-dominant PD as compared to postural instability gait difficulty subtype of PD (20). Neurologists should be aware of the difference in the NMS profile of ET and PD patients. While the NMS mentioned above in the context of ET can also be commonly observed in PD patients, there are several other NMS which are relatively more specific to PD. These include hyposmia, rapid eye movement sleep behavior disorder (RBD), dysautonomia, visual hallucinations, impulse control disorder, and constipation (21). Therefore, emergence of these NMS should prompt detailed evaluation to explore the possibility of PD or co-existent PD. The exact relationship between ET and PD is not well-understood but better understanding of the etiopathogenesis of ET and PD and their subtypes should lead to better insights into the relationships between these two common, but not well-defined movement disorders (10, 18).

Besides a link between ET and PD, there is a well-recognized link between ET-like phenotype and dystonia (see below discussion of tremor associated with dystonia). Several early studies have demonstrated that about 25% of patients with cervical dystonia had tremor in their hands that is phenomenologically similar to ET (22). In a more recent study of 2,362 patients enrolled in the Dystonia Coalition project, 53.3% had tremor, mostly involving the head, followed by the upper limbs and other body regions (23). Dystonic tremor (DT) occurred in 36.9–48.4% of patients, but others had ET-like tremors. The frequent co-existence of dystonia and ET-like tremor, and family history of both or either suggests that the two disorders share some pathophysiologic mechanisms, but the nature of the relationship is still poorly understood.

#### Pathogenesis of Essential Tremor

Although the exact pathogenesis of ET is still unknown and its detailed discussion is beyond the scope of this review, growing body of evidence suggest an alteration in the cerebello-thalamo-cortical circuit (24–26). While inferior olive was thought to play an important role in the pathogenesis of ET, a histopathological study of 14 ET patients did not reveal any abnormality compared to 15 control brains (27). Similarly, abnormalities of Purkinje cells have been observed in some (24, 25) but not (28) all post-mortem brain pathological studies. Hence, the cerebellar and olivary model of ET has remained controversial. Advanced neuroimaging studies have provided valuable insights into the putative neuroanatomical corelates of ET. Most of the studies based on structural or functional neuroimaging have identified abnormalities in the components of the cerebello-thalamo-cortical network, suggesting that ET might not be a disease associated with a particular brain region, rather associated with abnormalities in the neural network level (29).

Since a majority of ET patients have a family history of ET suggestive of an autosomal dominant transmission, attempts have been made to identify genetic abnormalities associated with ET. Although no single gene has been found to be causally linked to ET a number of genes (*ETM1, ETM2, ETM3, ETM4, ETM5, SORT1, SCN4A, SCN11A, HTRA2, CACNA1, SCNA, MTHFR, LINGO1, LINGO2, LRRK2, MAPT, TREMT, HMOX1, HMOX2; BACE2, LRRN2, DHRS13*, and *LINC00323*) have been identified in the last 3–4 decades as possibly related to ET (30). Further linkage, whole exome or genome sequencing, genome-wide association studies (GWAS), and other genetic studies are needed to elucidate the genetic mechanisms of ET.

#### Other Isolated Action Tremor Syndromes

As per the consensus statement, certain tremor syndromes may not fulfill the criteria of any of the established tremor syndromes and such cases should carry the label “indeterminate tremor syndrome” during the observation period (1). For example, isolated action tremor of both upper extremities with a duration <3 years (otherwise fulfilling the criteria for ET) should be labeled as “indeterminate tremor” during the observation period. Some of the isolated action tremor syndromes which get the label of “indeterminate tremor” may subsequently evolve and fulfill the definition of ET or may develop additional neurological signs and meet the diagnostic criteria of other diseases. For example, anoctamin 3 gene (*ANO3*) mutation which is known to cause an autosomal dominant cranio-cervical dystonia (DYT24) may initially present only with action tremor of upper extremities (31). Tremor in DYT24 commonly involves bilateral upper extremities and head; the tremor in extremities is usually asymmetric. As DYT24 was identified less than a decade ago, details about the natural course of the tremor and the exact neural correlates remain elusive. Patients with certain subtypes of spinocerebellar ataxias (SCA), especially SCA12 and SCA 40, may initially present with action tremor of the limbs, followed by the emergence of ataxia (32–34) (described in detail in a latter section). It is possible that the tremors observed in patients with DYT24, SCA12, and SCA40 are not completely “isolated” during the initial stages as the patients may have subtle dystonia and/or ataxia. Thus, the various tremor syndromes should be thoroughly investigated using accelerometry and electromyogram (EMG) as accurate distinction of these conditions may not always be possible solely by clinical examinations.

#### Isolated Rest Tremor

Rest tremor has been classically described in patients with PD; however, it has also been reported in ET patients, especially in those with a long duration of disease, and a variety of parkinsonian disorders. Suppression of rest tremor during initiation of voluntary movements of the affected body part usually indicates a state of dopaminergic deficiency such as PD. In a study on 44 PD patients and 22 ET patients, rest tremor suppression was observed in 39/44 PD patients and in 2/22 ET patients (35). As many of the patients with isolated rest tremor develop PD in the future, the term “benign tremulous parkinsonism” was used by several groups in the past (36, 37). Isolated rest tremor of at least 2 years duration was referred to as monosymptomatic rest tremor by the first consensus statement on tremor by the IPMDS (38). These patients should be followed closely because many subsequently develop additional signs of PD in the future (36). It should be noted that re-emergent tremor (discussed below) is viewed by some as a variant of rest tremor. Patients with dystonia may exhibit rest tremor in body parts not obviously affected by dystonia. Although this may possibly represent a form of dystonic tremor, when such rest tremor appears in a hand it may lead to a misdiagnosis of PD. In a study on 473 consecutive patients with adult-onset primary dystonia, 55.4% were tremulous and, of those, 40.7% had rest tremor (unilateral > asymmetric bilateral) (39). This observation highlights the fact that patients with isolated rest tremor should be thoroughly examined for additional signs of PD, ET, and dystonia.

It is important to note that to label rest tremor as “isolated,” the presence of subtle postural tremor should be ruled out objectively through accelorometry or surface EMG. In the absence of objective examination, it would be preferable to use the term “clinically isolated rest tremor.”

#### Enhanced Physiologic Tremor

As the name suggests, enhanced physiologic tremor can be observed in normal individuals during enhanced muscle activity such as while exercising or immediately thereafter, probably related to increased sympathetic activity. This is a form of action tremor which may not be visible to the naked eye because of its low amplitude and high frequency (8–12 Hz, slower in children and elderly) (1). Enhanced physiologic tremor usually involves both hands and all fingers symmetrically and is perhaps the most commonly observed postural tremor. Unilateral postural tremor mimicking enhanced physiologic tremor was reported in patients with reflex sympathetic dystrophy (40). If not very obvious to the naked eye, a sheet of paper may be placed on the outstretched hands to amplify the tremor to make it more evident (41). Vigorous exercise, fatigue, anxiety, stress, excess caffeine consumption, and conditions associated with a hypermetabolic state such as hyperthyroidism can make the enhanced physiologic tremor more obvious (42). Diagnosis of enhanced physiologic tremor is contingent upon the fact that other etiologies (axis-2 classification of consensus statement) of tremor are excluded. Considering the benign nature, this non-bothersome tremor usually does not warrant any pharmacotherapy. However, if bothersome, patients may obtain benefit from propranolol (2, 43). Similar to the isolated tremor syndromes described above, accelorometry and EMG can be used to confirm the nature of the tremor objectively. The objective confirmation of enhanced physiologic tremor requires the demonstration of the presence of tremor on both accelerometry and EMG (enhanced muscle activity via a recruitment of mechanical reflex loop i.e., both central and peripheral involvement) which cannot be demonstrated solely by clinical observation.

#### Isometric Tremor

This form of action tremor is observed when muscle forcefully contracts without moving the limb or the involved body part. For example, it is noted while holding a heavy object, while making a fist or tightly squeezing examiner's finger, or while contracting abdominal and truncal muscles when patient while seated flexes the hips and holds the legs against gravity (1). Isometric tremor may be isolated or noted in certain movement disorders such as PD, ET, orthostatic tremor, and dystonic tremor (44). Hence, individuals who exhibit isometric tremor should be thoroughly examined to explore the aforementioned disorders. There are two case reports of “shopping bag” tremor which phenomenologically is similar to isometric tremor (45, 46).

### Focal Tremors

The commonly reported focal tremors include voice tremor, head tremor, and palatal tremor, although the latter is also often referred to as palatal myoclonus since it is typically caused by rhythmical contractions of tensor veli palatine or levator veli palatine, rather than an oscillatory movement produced by antagonist contractions (see below).

#### Vocal/Voice Tremor

Vocal tremor or voice tremor (VT) occurs due to tremor of any of the anatomical components of the vocal apparatus. VT without any dystonia of the affected component of vocal apparatus or tremor in any other body part is referred to as isolated VT. Several studies have explored whether isolated VT is a unique category of tremor or a type of focal ET or a manifestation of laryngeal dystonic tremor (47, 48). VT results in periodic fluctuations in the pitch and loudness of voice, including voiceless pauses; the latter typically occurs as a compensatory phenomenon when vocalis muscles voluntarily contract in an attempt to suppress the VT. The latter is particularly common and troublesome when VT evolves into or becomes combined with laryngeal dystonia, also referred to as spasmodic dysphonia. Based on objective analyses of the VT of 160 subjects, one study reported that the normative frequency range of VT is 3.8–5.5Hz (49). A VT scoring system (VTSS), used to document the severity of VT based on a scale of 0–3 (maximum score 18), assesses six different components of the vocal apparatus (palate, the base of the tongue, pharyngeal walls, larynx, supraglottis, true vocal cords) (50). In addition to ET and laryngeal dystonia, VT may occur in the context of oro-facial dystonia and essential head tremor (HT), but it is relatively rare in patients with PD unless they also have co-existent ET (51, 52). A recent study based on the acoustic analysis of the voice of 240 subjects revealed the presence of VT in a number of neurological diseases with the following frequency- Huntington disease- 65%, ET- 50%, multiple system atrophy (MSA)-40%, cerebellar ataxia-40%, amyotrophic lateral sclerosis- 25%, progressive supranuclear palsy-25%, PD-20%, cervical dystonia- 10%, and multiple sclerosis-8% (53).

#### Head Tremor

HT is commonly seen in the context of ET and cervical dystonia. HT in the absence of any obvious cervical dystonia or any tremor of other body parts is described as isolated HT. Several studies have found that HT is often associated with cervical dystonia, neck pain, hand tremor and family history of tremor or other movement disorders, suggesting marked heterogeneity of underlying mechanisms (54, 55). In a series of 241 first-degree relatives of ET patients, isolated transient HT was observed in 21% (vs. controls 2%) which provides support for the observation that HT with or without hand tremor may be a manifestation of ET (56). In ET, based on the direction of the head movement, HT can be of 3 types- “Yes-Yes” (affirmation), “No-No” (negation), a mixed type, or “round-round” (diagonal) (57). In a series of 234 patients, HT was the presenting feature in more than two thirds of the patients (58). In the same study, ET patients with HT seem to have distinct characteristics as HT was often seen in the female patients, especially in those above 50 years of age (with a unimodal peak of age distribution), and patients with HT had a later onset of tremor (58). While this information support HT as a different “trait,” the increased prevalence of HT in patients with a long duration of ET also favors the concept that it could be both a “state” and “trait” dependent feature (59).

Several studies have drawn attention to HT in patients with cervical dystonia. Pal et al in a series of 114 patients with cervical dystonia observed HT in approximately two thirds of the patients; in one third HT was the presenting symptom (55). HT in cervical dystonia may be associated with the direction of pull resulting from dystonia and also with the duration of dystonia. There is discordance in the results of studies that explored the association of subtypes of cervical dystonia with the presence of HT. While a study on 185 patients with cervical dystonia reported that patients with retrocollis/anetrocollis had a higher likelihood of developing HT (60), another study on 293 patients reported that torticaput variety of cervical dystonia is more likely to be associated with HT (61). Duration of dystonia was the common factor related to HT in both these studies. Similar to that ET, there is evidence to suggest that HT in cervical dystonia has some unique features. In a large multi-center study comparing the clinical characteristics of tremulous (HT at disease onset) and non-tremulous cervical dystonia patients, the former group more frequently affected older women, had a higher prevalence of ataxic features and had milder dystonia (62). A structural imaging study revealing greater cerebellar vermian atrophy in cervical dystonia patients with HT compared to those without HT further reinforces the fact that HT represents a unique cerebellar phenotype of cervical dystonia (63). One characteristic feature that helps to differentiate between HT due to cervical dystonia vs. ET is the presence of “null point,” a position of the head and neck when the head tremor diminishes or resolves as the head and neck are allowed to assume the maximal dystonic position (64). Assessment of tremor in the supine position may provide a clue toward the nature of HT. HT in patients with ET tends to disappear in supine position whereas HT associated with cervical dystonia persists in the supine position and may be associated with the abnormal dystonic posture (65).

#### Palatal Tremor

This is a rare form of tremor that involves the soft palate. It was previously known as “palatal myoclonus” but it was renamed “palatal tremor” during the first International Congress of Movement Disorders in 1990 as the term “tremor” represents the continuous, rhythmic nature of the palatal movement (66). However, the term myoclonus may still apply since the movement is produced by contractions of only agonist muscles (either tensor veli palatine or levator veli palatine), rather than alternating, oscillatory antagonist contractions which produce typical oscillatory movement characterizing tremor. Furthermore, in contrast to typical tremor, this focal movement disorder often has a jerky and arhythmic component, particularly when present as a functional (psychogenic) movement disorder (67, 68).

Based on the absence or presence of additional neurological signs and symptoms palatal tremor is categorized into two groups, essential palatal tremor (EPT), and symptomatic palatal tremor (SPT). EPT, in a true sense, is an isolated focal tremor as the sole manifestation of this entity is palatal tremor, often with audible clicks. The clicks are presumably secondary to rhythmic contraction of tensor veli palatini muscle. No demonstrable etiology is found in patients with EPT. The frequency of EPT may vary from <1 to 7 Hz (69). SPT, which is more frequently reported compared to EPT, refers to the conditions where palatal tremor coexists with other neurological signs and symptoms. SPT is reported to have lower frequency than that of EPT, in the 1.5–3 Hz range, and may be associated with myorhythmia (see below) involving other head and neck structures (69). While EPT may have complete cessation during sleep, SPT usually persists during sleep, albeit with a lower frequency (69, 70).

In addition to functional (psychogenic) palatal tremor (71), there are many other etiologies. Previous case series have documented vascular abnormalities (posterior circulation strokes, aneurysms, arterio-venous malformation), genetic abnormalities (polymerase gamma-related mitochondrial disease, SCA type 20, Alexander disease), and traumatic brain injury as the commonest etiologies of SPT (67, 72). In addition, there are reports of an array of neurodegenerative (progressive ataxia with palatal tremor), infectious (Whipple disease, tuberculosis, toxoplasmosis), inflammatory/demyelinating (neurosarcoidosis, multiple sclerosis, Behcet's disease) and neoplastic conditions (posterior fossa tumors) associated with SPT (67, 72, 73). Although not universal, MRI of the brain often reveals hypertrophic degeneration of the olive and other focal lesions in the Guillain-Mollaret triangle (formed by the ipsilateral red nucleus, inferior olivary nucleus, and contralateral dentate nucleus).

### Task-Specific Tremor

Task-specific tremor is a type of action tremor that emerges while performing or attempting to perform specific motor tasks such as writing and playing musical instruments. Primary writing tremor (PWT) is one of the commonly reported task-specific tremors. It is described as a tremor of the hand only while writing or while attempting to write (74). Based on the timing of the tremor, PWT is divided into two categories- type-A (tremor while actively writing) or type-B (tremor while adopting the hand position used for writing). Hence, type-B PWT is a position-specific tremor rather than a true task-specific tremor (74). Although PWT affects the hand used for writing which is often the dominant hand, it may subsequently affect the other hand also (75). The abnormal movement or position in the opposite, unaffected, hand may be observed as a mirror dystonia or tremor (76). The frequency of PWT is 5–7 Hz and it often has a jerky component (77). Etiopathogenesis of PWT remains elusive. Several structural and functional neuroimaging studies have suggested a putative role of the cerebellum in the genesis of PWT (78, 79). Although it has been categorized as “tremor,” there is controversy whether PWT is truly an isolated tremor or it is a dystonic tremor associated with the writer's cramp (80, 81). Electrophysiological assessment comparing several characteristics of PWT and dystonic tremor (DT) provided evidence for marked similarity of these two conditions in several electrophysiologic indices, including reduced eyeblink classic conditioning learning, reduced blink recovery cycle inhibition, and a lack of effect of paired-associative plasticity on long-interval intracortical inhibition (82). While additional studies are warranted to confirm and establish these findings, these findings certainly reinforce the notion that PWT is a phenotype of task-specific dystonia.

Many examples of task-specific tremor have been reported, including task-specific tremors in musicians (83, 84), oro-lingual tremor only while drinking (85, 86), chin tremor only while brushing teeth (87), finger tremor in carrom players (88) and many others. Patients with task-specific tremor should be followed up periodically to assess the emergence of additional neurological signs. This is important as there are reports to suggest that some of these patients subsequently develop PD (89, 90). In a recently published case series, 11 patients with various types of task-specific tremor of the arm went on to develop PD with a mean duration between onset of task-specific tremor and the onset of PD 13.66 ± 14.36 years (89).

### Orthostatic Tremor

Orthostatic tremor (OT) refers to a high-frequency (13–18 Hz) tremor of the legs upon standing. Rarely, trunk and abdomen may be involved. When OT is the only clinical feature, i.e., an isolated tremor syndrome, it is termed a primary OT. The key phenomenological characteristics include high frequency, low amplitude tremor when the individual stands up and tremor resolves immediately after sitting or lying down (91). Very low amplitude and high frequency of OT may not be often obvious to the eyes and in such cases, surface EMG may be useful. Hence, for an accurate correct axis I classification of OT objective physiological assessment should be performed. Palpation and auscultation of the leg muscles may reveal the presence of thrill, and a continuous thumping sound (Helicopter sign), respectively (91). Most of the patients with OT report subjective unsteadiness and/or cramp in the distal legs upon standing and recent studies also provide objective evidence of ataxia in patients with OT (92). The mechanism of subjective unsteadiness in OT is not well-understood but has been attributed to a tremulous disruption of the proprioceptive feedback from the lower limbs (93). It is not clear whether the disruption is altered by trans-spinal direct current stimulation, which has been recently found to provide modest improvement in OT (94). The term “OT plus” is used to describe a situation when OT co-manifests with additional neurological conditions such as parkinsonism, ataxia, dementia. In a recently published series of 27 patients with OT, neurological comorbidities preceding the onset of OT were present in 30% of the patients (95). The exact etiopathogenesis of OT is unclear and several hypotheses which include altered cerebello-thalamo-cortical circuit, cerebellar neurodegeneration, dopaminergic deficit, and presence of a central oscillator have been proposed (91). OT must be differentiated from other leg tremors, including leg tremors present in patients with ET or PD (96).

Although 13–18 Hz tremor is characteristic of OT, there are several reports of OT with frequency <13 Hz (slow OT). There are reports of slow OT as an isolated syndrome as well as in the context of other neurological disorders (97, 98). A retrospective analysis of 28 patients revealed the presence of slow OT (<13 Hz) in 14 patients and among 8 of those with slow OT had a tremor frequency of <10 Hz (97). Interestingly, low (<10 Hz) and intermediate frequency (10–13 Hz) of OT in the same study were associated with more subjective unsteadiness, abnormal gait examination, and falls (97). Slow OT is also referred as to pseudo OT and in addition to fast OT (99), it has also been reported in patients with ET and PD (100, 101).

## Tremor in the Setting Additional Neurological Features

### Tremor Associated With Parkinsonism

#### Rest Tremor

Tremor-at-rest or rest tremor is one of the hallmark clinical features of PD. In a study on autopsy-confirmed PD cases, 69% had rest tremor at the time of presentation and 75% had it during the course of the disease (102). Rest tremor in PD is typically asymmetric, has a frequency of 4–6 Hz, commonly involves the hands, in a “pill-rolling” pattern, but may involve other body parts, and is often exacerbated during walking or while performing physical or mental tasks. Inhibition of the tremor during voluntary movements is a characteristic feature of rest tremor in PD (103). There are several paradoxical aspects of rest tremor in PD including lack of correlation with the degree of nigrostriatal degeneration, occasional occurrence on side contralateral to predominant parkinsonian features (bradykinesia/rigidity), resolution of rest tremor in some patients with progression of disease, and inconsistent response to levodopa (104, 105). Although the accurate neuroanatomical corelates of rest tremor is yet to be fully understood, there is evidence suggesting that both basal ganglia and cerebello-thalamo-cortical circuits are involved in the generation of rest tremor (104).

#### Re-Emergent Tremor

The term “re-emergent tremor” was coined by Jankovic et.al (106) to describe a form of postural tremor in patients with PD that emerges after a latency of a few seconds when hands and arms are held in an anti-gravity horizontal posture. The readers are referred to published video demonstration of the examination for re-emergent tremor (107). Although most often re-emergent tremor coexists with observable rest tremor, it may rarely emerge independently in PD patients without rest tremor (108). Previous cross-sectional studies have documented re-emergent tremor in 20–25% of PD patients (109, 110). An EMG study exploring the nature of postural tremor in PD revealed two pathophysiologically distinct clusters: 81% had re-emergent tremor and 19% had a pure postural tremor (111). The exact neural correlates of re-emergent tremor remain elusive; however, there is evidence to suggest that it overlaps with parkinsonian rest tremor in terms of frequency (both are of 3–5 Hz), the direction of movement (occasional supination-pronation), and response to dopaminergic medications (106, 111, 112). A recent study based on transcranial magnetic stimulation demonstrated that re-emergent tremor and rest tremor have common pathophysiological mechanisms in which the motor cortex plays an important role (113). The amplitude of re-emergent tremor and the tremor pause duration (latency) was demonstrated to have an inverse relationship and both are also modulated by levodopa (114). Amplitude and latency are also affected by provocative measures or distractions as noted by increase in amplitude and a decrease in latency when the patients count out loud backward from 100 (115). Patients with PD may rarely present with re-emergent tremor of the tongue (116–118). Re-emergent tongue tremor has also been reported in conditions other than PD (119, 120). Re-emergent tremor of the jaw was reported both in idiopathic PD (121) and vascular parkinsonism (122). Re-emergent tremor was also described while drawing a spiral (123).

### Dystonic Tremor and Tremor Associated With Dystonia

Dystonic tremor (DT) represent a condition where dystonia is the predominant neurological feature and tremor manifests in the body part associated with dystonia. If a patient with dystonia has a tremor in a non-dystonic body part, the tremor is described as “tremor associated with dystonia” (TAWD) (15). For example, a hand tremor in a patient with cervical dystonia would be classified as TAWD. Occasionally, patients may develop DT as well as TAWD (124). While DT can affect any body part, it is most frequently found in patients with cervical dystonia (as head tremor) (124–126). The onset of DT usually either coincides with or occurs after the onset of dystonia. Rarely, DT may precede the onset of dystonia (127). One of the key features of DT is irregularity and variability in the frequency and amplitude. DT can be of postural, kinetic, or rest in nature and can manifest with varied combination of these phenomenologies (39) (Figure 1). DT can be reduced or completely eliminated by alleviating maneuver (128, 129), also referred to as “sensory trick” “geste antagoniste,” and when the affected body part is positioned in the direction of dystonia and the movement, tremor or abnormal posture stop, this is referred to as “null point” (64). Conversely, the severity of DT worsens with the voluntary orientation of the affected body part against the main direction of dystonia pull (e.g., a patient with right torticollis may have an increase in DT while turning the head to the left or while trying to maintain primary head position) (130).

### Holmes Tremor

Holmes tremor was first described by Gordon Holmes in reference to a 3–4 Hz tremor which is usually of high amplitude, irregular, present at rest, worsens with posture, and additionally intensifies with action (131). Holmes tremor predominantly affects the proximal upper extremities unilaterally or asymmetrically. There are several synonyms for Holmes tremor, including rubral tremor, thalamic tremor, midbrain tremor, mesencephalic tremor, and cerebellar outflow tremor (131, 132). Holmes tremor almost always occurs in the context of pathologies in the brainstem or diencephalon. A recent connectivity-based study analyzed the pattern of structural pathology in previously published case reports and suggested that the affected brain legions are connected to a common brain circuit with nodes in the red nucleus, thalamus, globus pallidus, and cerebellum (133). As per the new “consensus” tremor classification (1), it is one of the tremor syndromes which is associated with additional neurologic signs. In a series of 29 patients, the common co-existing neurologic abnormalities were hemiparesis (62%), ataxia (51.7%), hypoesthesia (27.6%), and dystonia (24.1%) (132). While stroke and traumatic brain injury are leading causes of Holmes tremor (132), it has also been reported in patients with multiple sclerosis (134), brain tumor (135), intracranial hypotension (136), and CNS infections (137). There may be a latency of a few weeks to few years between the precipitating events and the onset of the tremor. Holmes tremor may be associated with myorhythmia (below). A recently published study on 17 patients with Holmes tremor suggested the existence two phenotypically distinct types of Holmes tremor i.e., midbrain Holmes tremor and thalamic Holmes tremor (138). While the former was characterized by myorythmic rest tremor with or without distal dystonic posturing, the latter had distal choreo-athetoid movements, marked dystonic posturing, and proprioceptive sensory deficits.

### Myorhythmia

As per the consensus paper, myorhythmia is classified as a tremor syndrome with prominent additional signs (1). The term “myorhythmia” was first coined by Herz in 1931 in reference to a slow tremor in a patient with dystonia. This is an uncommon movement disorder which is characterized by slow, rhythmic, repetitive jerky movements of 1–4 Hz frequency, involving the cranial or limb muscles (1, 70). It is frequently associated with other neurological signs such as dystonia, palatal tremor, and eye movement abnormalities, and can affect cranial, branchial and limb muscles along with the additional neurological signs (70). Rarely it can manifest as isolated facial slow rhythmic movement (139).

The precise neural mechanism of myorhythmia remains elusive but the main significance of recognizing this movement disorder is that it is almost always associated with an identifiable pathology typically involving the upper brainstem and thalamus. Myorhythmia has marked etiological heterogeneity. It has been frequently reported as oculo-masticatory myorhythmia in the context of Whipple's disease, caused by the infection of the central nervous system by *Trophyrema whipplei* (140). Other conditions where myorhythmia has been reported are stroke (139), anti-NMDA encephalitis (141, 142), anti-IgLON5 disease (143), interferon alpha-2a use (144), Hashimoto encephalopathy (145), and X-linked dystonia-parkinsonism (146). As myorhythmia is often associated with conditions that are potentially treatable, it is important to be familiar with this phenomenology and its differential diagnoses.

### Wing Beating Tremor

This form of tremor often overlaps with Holmes tremor. It has been classically described in patients with Wilson's disease (WD), but there are many other forms of tremor associated with WD. Wing beating tremor is a low frequency, high amplitude postural tremor which is usually elicited by sustained abduction of the arms with flexed elbows and palm facing downwards (147). Considering frequent association with WD, patients with this form of tremor should be thoroughly investigated for WD. It usually coexists with several other neurological signs such as dystonia, Kayser-Fleischer ring in the cornea, cognitive impairment in patients with WD (148). Wing beating tremor was reported recently in a case of Creutzfeldt-Jakob Disease (CJD) (149).

## Functional Tremor

Functional or psychogenic tremor is the most commonly reported functional movement disorder, accounting for approximately half of the cases (150, 151). There are no set diagnostic criteria for functional tremor and the diagnosis is based on a careful history and neurological examination. Among the commonly described characteristics of functional tremor are variability, distractibility, entrainability, and coherence; and higher prevalence in females compared to males (152). The onset of tremor is usually sudden and there is variability in the amplitude, frequency, and direction of the tremor. In one study designed to determine which clinical features help distinguish functional tremor from ET, a “blinded” rater evaluated video segments of subjects using a standardized protocol with special attention to distractibility, suggestibility, or entrainment (153). Patients with functional tremor were significantly more likely to have sudden onset, spontaneous remissions, shorter duration of tremor, and lower prevalence of family history of tremor. Furthermore, patients with functional tremor had more distractibility with alternate finger tapping and mental concentration, suggestibility with a tuning fork, and exacerbation with hyperventilation. Although functional tremors can affect any of the body parts, hands are the most commonly reported body involved in functional tremor. A tremor in multiple body parts occurring with similar frequencies (coherence) is a clue toward functional tremor. Careful assessment of these features may help in distinguishing functional tremor from other common diseases presenting with tremor including ET and PD (152). Electrophysiological assessment, using a scoring system, may provide additional information to support the diagnosis of functional tremor (150, 154). However, it needs to be emphasized that the positive signs on the clinical examination mentioned above are the key to the diagnosis of functional tremor. The readers are referred to two published articles with video demonstration of examination of functional tremor (152, 155).

## Other Rare Forms of Tremor

In this section, we describe some of the rare axis-2 tremor syndromes which are likely to be encountered in the general neurology and movement disorders practice, often on the background of other neurological problems or movement disorders.

### Neuropathic Tremor

A neuropathic tremor is a form of tremor observed in some patients with severe peripheral neuropathies in the absence of any other movement disorders (156). Certain peripheral neuropathies, especially demyelinating polyneuropathies, have a higher predilection than other neuropathies for neuropathic tremor. The commonly described tremor frequency is 3–6 Hz, it usually affects the arms and/or hands, and does not vary with weight loading (156). In a series of 89 patients with polyneuropathy, 59.5% during clinical evaluation and 74% during objective assessment through surface EMG recording were noted to have tremor (157). Postural tremor (70%) was the commonest, followed by rest (51%) and kinetic tremor (32%). A study on 43 patients with inflammatory neuropathies revealed that tremor was most common in IgM paraproteinemic neuropathies, followed by chronic inflammatory demyelinating polyradiculoneuropathy (CIDP), and multifocal motor neuropathy with conduction block (158). Several studies have reported that patients with a specific subtype of CIDP which is associated with the presence of neurofascin155 (nfasc155) IgG4 antibodies develop disabling low-frequency, high-amplitude action tremor of the upper limbs (159, 160). Head, voice, and tongue tremor have also been reported in this subtype of CIDP (161, 162). There are several reports of a high prevalence of tremor in patients with various forms of Charcot-Marie-Tooth disease (CMT), in the past referred to as the Roussy-Levy syndrome (163, 164). In a survey of 201 patients, 40% of the CMT patients reported the presence of tremor of hands (164). Because of frequent involvement of hands, presence of postural tremor, presence of a family history of ET, and lack of correlation of tremor severity with neuropathy severity, it was presumed that tremor in CMT may pathophysiologically overlap with that of ET. A study based on the neurophysiological evaluation, however, did not find any evidence of cerebellar dysfunction in CMT patients with tremor (165).

### Tremor in Spinocerebellar Ataxias

Various ataxias may be also associated with tremor. For example, SCA 12 is an autosomal dominant progressive degenerative ataxia that is commonly reported among the “Agarwal” community in northern India (32). SCA12 is due to the abnormal CAG repeats expansion in the 5' untranslated region of PPP2R2B gene at locus 5q32. The most common presenting symptom of SCA12 is action tremor of both upper extremities, often misdiagnosed as ET. Subsequently, patients develop appendicular and gait ataxia. In a series of 21 consecutive patients, postural tremor was observed in 17 patients (81%), followed by head tremor in 13 (62%), intention tremor in 12 (57%), and rest tremor in 10 (48%) (34). Upper extremity tremor in SAC12 is slow compared to that in ET and has more proximal involvement. A recent study noted the presence of action tremor in all and an asymmetry of the tremor amplitude in 91% of the patients with SCA12 (166). A patient with SCA40 was reported to have an ET-like syndrome for years, requiring treatment with deep brain stimulation, before the genetic cause was confirmed (33).

### Fragile-X Tremor Ataxia Syndrome

FXTAS is a neurodegenerative disorder that results due to CGG repeat expansion in the premutation range (55–200) in the fragile X mental retardation 1 gene (*FMR1* gene) (167). Tremor and ataxia are the predominant clinical features along with a repertoire of other symptoms that include cognitive dysfunctions, parkinsonism, peripheral neuropathy, anxiety, depression, and apathy (168). Although action tremor in both upper limbs is the common type of tremor in FXTAS, patients may also have rest tremor (169). Because of a mixed phenomenology of tremor along with mild parkinsonian signs, FXTAS may be confused with ET or PD. However, the presence of early ataxia and cognitive impairment usually differentiates it from ET or PD. Previous studies have reported a correlation of the CGG repeat length with the onset of the motor symptoms.

#### Other Genetic Forms of Tremor

Klinefelter syndrome (47, XXY) (KS) is a chromosomal variation leading to the presence of an extra X-chromosome in males (170). Patients usually have an array of symptoms related to endocrine, metabolic, and reproductive functions. Commonly reported features include tall stature, micro-orchidism, gynecomastia, azoospermia, sparse body hair, and osteoporosis (170). There are several reports of a high prevalence of tremor in patients with KS. In a series of 44 patients with KS, more than half (51%) of the patients reported tremor, and 10% were previously diagnosed as ET (171). Although bilateral or unilateral action tremor of the upper extremities is commonly reported, some patients may present with rest tremor (172). The exact pathogenesis of tremor in KS is not fully understood.

Spinal and bulbar muscular atrophy or Kennedy disease, a rare X-linked neuromuscular disease caused by a CAG repeat expansion in the first exon of the androgen receptor gene, is manifested by bulbar symptoms, muscle cramps, leg weakness, and tremor (173). The patients have evidence of small or large nerve fiber neuropathy and, therefore, the observed tremor may be a neuropathic tremor.

Hereditary chin tremor (HCT), also known as hereditary geniospasm, hereditary quivering of the chin, hereditary essential chin myoclonus, is a benign genetic condition which manifests only with chin tremor. HCT is linked to chromosome 9q13-q21 (174). It follows autosomal dominant transmission and has high penetrance. Chin tremor may be visible in patients with HCT from childhood and it peaks during early adulthood. One of the key features of HCT is the intermittent nature of the tremor that is triggered by emotional stress or anxiety and lasts for few seconds to a few hours. The frequency of HCT varies from 2 to 11 Hz (175). This disease is usually non-progressive and does not have any long-term complications. It can be effectively treated with local injections of botulinum toxin (176).

There are several other genetic disorders that may have tremor as one of the clinical features (177), but detailed discussion of all the those syndromes is beyond the scope of this article.

## Current Controversies

### ET Plus- the Controversial Category

One of the most recently debated issues in the field of tremor is the introduction of the term ET plus by the “consensus” statement (1). As discussed earlier, ET with additional neurological soft signs is now labeled as ET plus, as per the new tremor classification. This categorization has its own merits and limitations (10). The classification defines isolated ET which is helpful for genetic studies and for selection of a homogenous population of patients in interventional trials. However, the presence of poorly defined “soft signs” is troublesome. For example, “questionable dystonia” assessed by one neurologist may not be clinically obvious, bringing in the risk of inter-rater variability (178, 179). Hence, a “soft sign” for one examiner may be a “no sign” for another examiner or a separate and distinct disorder for another examiner. This uncertainty about the presence and relevance of such “soft” signs makes the classification challenging. Therefore, in the absence of reliable objective biomarkers, an accurate clinical distinction between ET and ET plus, only on the basis of these subtle/questionable signs may not be possible. While it needs to be confirmed by additional studies, a recent post-mortem study that compared certain pathological changes in the cerebellum of ET and ET plus patients did not find any significant difference between the two conditions (180). The introduction of ET plus group will have substantial impact on epidemiological studies. Indeed, since the publication of the Consensus statement, many studies have demonstrated that ET Plus is more prevalent than ET (6–10). In such scenarios, the significance of the previous clinical and epidemiological studies in which a large proportion of ET plus patients were categorized as ET, is going to be relatively uncertain (8). Additionally, as ET plus is a time-sensitive diagnostic placeholder, counseling the patients about the diagnosis and the expected clinical course is going to be challenging.

### ET- a “Disease” or “Syndrome?”

Before the introduction of the new tremor classification by the IPMDS, ET has been regarded as a “disease” or a “family of diseases” (181, 182). However, the new classification describes ET as a tremor “syndrome.” This change has stimulated scientific debates as to whether ET should be regarded as a “disease” or a “syndrome” or whether the various variants of ET should be simply considered subtypes, such as ET-PD, ET-dystonia, ET-ataxia, and other, since one may with time evolve into another (10, 183–185). A recent study using multimodal investigations, including objective gait assessment, neuropsychological assessment, and optical coherence tomography (OCT) for retinal thickness measurement, provided objective evidence for the existence of two ET subtypes (186). Using cluster analysis one subtype, characterized by midline tremor, cognitive decline and thin retinal inner layer, suggests that this subtype of ET is more likely to be associated with neurodegeneration. Hence, additional studies exploring and confirming the existence of such ET subtypes would provide more scientific insight to this “disease vs. syndrome” controversy.

## Future Perspectives

While there has been a substantial progress in the research on pathophysiology of ET, the exact neural correlate still remains elusive. Majority of the studies, as mentioned above, indicate structural and functional abnormalities in the cerebellum (especially in the Purkinje cell) and in the cerebello-thalamo-cortical circuit. However, these studies have not yielded any objective biomarkers for ET that can supplement the clinical diagnosis at an individual level. Therefore, future studies should explore more data-driven approach to utilize multi-modal imaging and electrophysiology to supplement the clinical diagnosis of ET.

The introduction of the term “ET plus” by the “consensus statement on the classification of tremors” (1) generated much controversy and numerous publications. When applied in clinical practice many (perhaps most) patients with prior diagnosis of ET now have to be reclassified as “ET-plus.” Furthermore, when followed prospectively many patients with ET evolve into “ET plus.” Hence, longitudinal studies of patients with isolated (“pure”) ET are needed to determine which characteristics of the tremor, or associated “soft signs,” predispose some patients to transition to “ET plus.”

Future research should also address other issues related to the diagnosis and classification of tremor syndromes. For example, two common features of ET, the presence of family history of ET and alcohol responsiveness, were not included in the diagnostic criteria of ET in the new classification of tremor (1). It would be interesting to see if these two features are predictive of future outcome or a particular subtype of ET. Thus, the entity of isolated ET should be considered a time-sensitive diagnostic placeholder.

PWT should be another fruitful area of research in the future. It has been debated for long time whether it is a distinct entity or a variant of ET or dystonic tremor. As discussed above, a recent study has provided compelling evidence in support of important dystonic component to this form of tremor (82). If confirmed through additional multimodal diagnostic interventions, terms such as “dystonic writing tremor” or “writers' dystonic tremor” would more accurately reflect the underlying dystonia. As cerebellar abnormalities have been reported in studies on PWT (91), the concept that PWT is dystonic in origin would pave the way for additional research on the role of cerebellum in the pathogenesis of dystonia and dystonic tremor (187, 188).

One of the tremor syndromes which needs more clarity and consensus on the nomenclature is “palatal tremor.” Although currently described as “tremor,” it clearly does not fit into all the characteristics of tremor and it phenomenologically overlaps with segmental myoclonus and myorthythmia.

**Figure 2:**
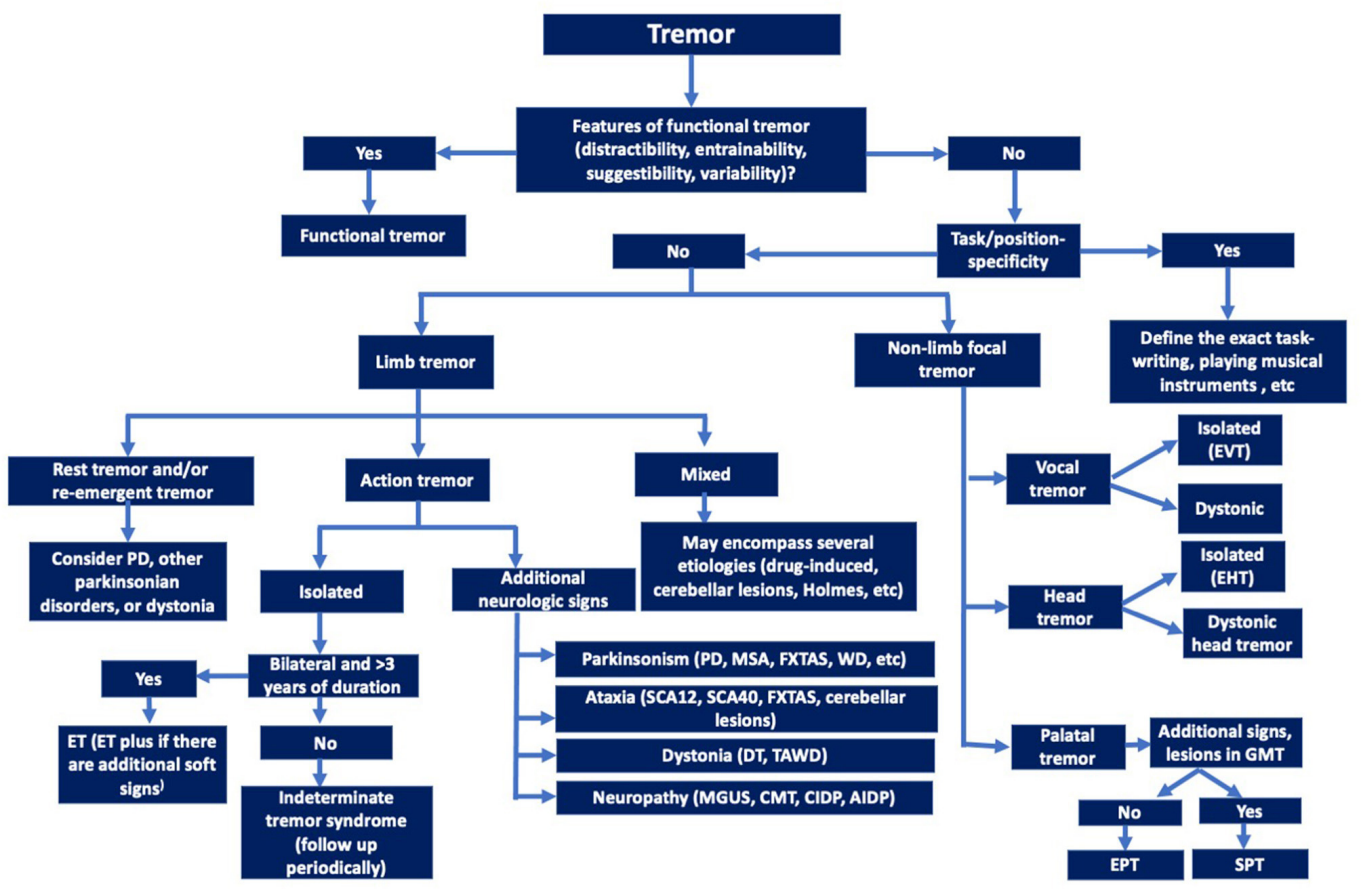
Decision tree for clinical identification of the major tremor syndromes (Axis-2 etiologies should be explored for all the tremor syndromes). EVT, Essential vocal tremor; EHT, Essential Head tremor; EPT, Essential palatal tremor; SPT, Symptomatic palatal tremor; ET, Essential tremor; PD, Parkinson's disease; DT, Dystonic tremor; TAWD, Tremor associated with dystonia; MSA, Multiple system atrophy; FXTAS, Fragile-X-tremor ataxia syndrome; CMT, Charcot-Marie-Tooth disease; MGUS, Monoclonal gammopathy of uncertain significance; AIDP, Acute inflammatory demyelinating polyradiculoneuropathy; CIDP, Chronic inflammatory demyelinating polyradiculoneuropathy.

Ultimately, better understanding of physiological, genetic, pathological and other biological mechanisms is critical for development of diagnostic biomarkers that would facilitate classification and subtyping of tremors (Figure 2) and eventually leading to pathogenesis-targeted therapies.

## Author Contributions

AL: design and conceptualization of the work, prepared the first draft of the manuscript. JJ: design and conceptualization of the work, critical review, and editing of the manuscript. All authors contributed to the article and approved the submitted version.

## Conflict of Interest

The authors declare that the research was conducted in the absence of any commercial or financial relationships that could be construed as a potential conflict of interest.

## Publisher's Note

All claims expressed in this article are solely those of the authors and do not necessarily represent those of their affiliated organizations, or those of the publisher, the editors and the reviewers. Any product that may be evaluated in this article, or claim that may be made by its manufacturer, is not guaranteed or endorsed by the publisher.
